# Correlation of Albuminuria and Diabetic Retinopathy in Type-II Diabetes Mellitus Patients

**DOI:** 10.7759/cureus.21927

**Published:** 2022-02-05

**Authors:** Shovna Dash, Abhilash Chougule, Soumyakanta Mohanty

**Affiliations:** 1 Ophthalmology, Kalinga Institute of Medical Sciences, Bhubaneswar, IND

**Keywords:** nephropathy, macroalbuminuria, microalbuminuria, retinopathy, diabetes

## Abstract

Background: Diabetic ocular disease is a leading cause of blindness today. The most common microvascular complications of diabetes are diabetic retinopathy and diabetic nephropathy. Multiple risk factors like the duration of the disease, age of the patient, high blood pressure, pregnancy, blood glucose control, and nephropathy have been studied to be associated with the development and progression of diabetic microangiopathy. However, the association of albuminuria has still not been studied in detail, especially in type-II diabetes mellitus.

Aim: The primary objective of our study is to quantify the relationship between diabetic retinopathy and urine albumin excretion and to correlate the urinary albumin excretion (normoalbuminuria, microalbuminuria, macroalbuminuria) with the severity and grade (mild, moderate, severe non-proliferative diabetic retinopathy [NPDR] or proliferative diabetic retinopathy [PDR]) of diabetic retinopathy.

Methods: In this cross-sectional study, 250 patients with type-II diabetes above 40 years of age attending the ophthalmic outpatient department (OPD), Kalinga Institute of Medical Sciences (KIMS), Bhubaneswar in India between September 2019 and September 2021 were subjected to a detailed evaluation of history and a thorough ocular examination. Besides, a blood sugar estimation and urine albumin levels were documented. The grade of diabetic retinopathy was correlated with albumin levels.

Results: The duration of diagnosis of diabetes ranged from 1-25 years. The association between the grade of diabetic retinopathy and the duration since diagnosis was significant. Sixty-nine percent of the cases were hypertensives, and 66.7% of hypertensives had diabetic retinopathy. In patients without retinopathy, 83.03% had normoalbuminuria levels, and 16.96% had microalbuminuria. In the mild NPDR group, 37.94% of cases had normoalbuminuria, and 62.06% had microalbuminuria. In the moderate NPDR group, 11.1% of cases had normoalbuminuria, and 88.8% had microalbuminuria. In the severe NPDR group, 57.14% of cases had microalbuminuria, while 42.86% had macroalbuminuria. In the very severe NPDR group, 42.86% of cases had microalbuminuria, and 57.14% had macroalbuminuria. In the PDR group, only 6.6% of cases had microalbuminuria, and the rest, 93.3%, had macroalbuminuria.

Conclusion: This study concluded that there is a definite association between albuminuria and severe diabetic retinopathy in type-II diabetes. Microalbuminuria was a finding associated with all grades of retinopathy with skewing towards the lower grades of diabetic retinopathy; a proportion of diabetics without retinopathy also had microalbuminuria, while macroalbuminuria was associated only with those patients who had either severe NPDR, very severe NPDR, or PDR.

## Introduction

Diabetes is one of the largest global health emergencies of the 21^st^ century [1]. The world has around 246 million people with diabetes [2]. Diabetes has reached epidemic proportions in India in the 21^st^ century, with 65.1 million people suffering from diabetes [3].

Diabetes is characterized by metabolic abnormalities and long-term microvascular and macrovascular complications. While there are many risk factors associated with the development and progression of retinopathy, the duration of the disease and the age of the patient are said to be the most predictable. Several other factors, including high blood pressure, pregnancy, blood glucose control, and nephropathy, have been linked with a greater risk for developing diabetic microangiopathy. The role of microalbuminuria, dyslipidaemia, body mass index (BMI), and smoking as predictors of diabetic retinopathy is not well understood.

Diabetic retinopathy (DR) and diabetic nephropathy (DN) (now called diabetic kidney disease [DKD]) are the most common microvascular complications of diabetes. DR and DKD are major causes of social and economic burden to individuals with diabetes and the healthcare system due to the risk of blindness [3] and end-stage renal disease [4]. Diabetic retinopathy is the most common and probably the most serious of all ocular problems. Due to its microvascular complications, diabetic retinopathy accounts for 4.8% of the 37 million cases of blindness in the world [2].

DR is considered as a sign of generalized microangiopathy that occurs in a diabetic patient. Microalbuminuria signifies a minor quantity of albumin ranging between 30 mg/24 hours to 300 mg/24 hours to pass out of renal filtration barrier to appear in urine, while macroalbuminuria is more than 300mg/24hrs [5]. Congruence between microalbuminuria and retinopathy has been well reported in persons with type-I diabetes, and a lesser number of studies address the association between microalbuminuria and type-II diabetes [6,7].

In this study, we aim to quantify the relationship between diabetic retinopathy and urine albumin excretion and to correlate the urinary albumin excretion (normoalbuminuria, microalbuminuria, macroalbuminuria) with the severity and grade (mild, moderate, severe NPDR or PDR) of diabetic retinopathy.

## Materials and methods

This cross-sectional study was conducted in the Department of Ophthalmology, Pradyumna Bal Memorial Hospital, KIMS, Bhubaneswar, between September 2019 to September 2021, with a sample size of 250 patients. Ethical clearance from IEC KIMS (KIMS/KIIT/IEC/108/2019/6.9.2019) and consent from the patients was obtained before enrolment of the cases. Patients aged 40 years or more with type-II diabetes mellitus - established cases (on anti-diabetic medications)/recently diagnosed cases (defined by American Diabetes Association) [6] were included in the study. Patients who were excluded were those having significant media opacities precluding fundus examination and those who have been treated earlier with either light amplification by stimulated emission of radiation (LASER) or intravitreal anti-vascular endothelial growth factor (VEGF) injections. Patients with essential hypertension, with pre-existing renal diseases like renal failure, obstructive uropathy, urinary tract infections, interstitial nephropathy, glomerulonephritis were excluded as well.

Informed written consent was obtained in every case. A detailed ocular history and medical history were taken. A detailed general physical examination was performed. Blood pressure was recorded in all the cases - a total of three readings were taken. All recordings were done with the patient in a sitting position. The patients were given rest of 15 minutes before each blood pressure recording. The average of three recordings was taken as the final value. Patients who were already on anti-hypertensive medications and those with a systolic blood pressure of more than 140 mm Hg and/or diastolic blood pressure of more than 90mmHg were taken as hypertensives according to clinical practice guidelines for high blood pressure [8].

Visual acuity was recorded for both distance and near, and best-corrected visual acuity (BCVA) was recorded. Intra-ocular pressure (IOP) was recorded using a non-contact tonometer. An elaborate biomicroscopic examination of the anterior segment was performed. Pupils were dilated with topical medication of 1% tropicamide and 5% phenylephrine drops, the latter being omitted in hypertensives. Detailed fundoscopy was done by direct ophthalmoscopy, indirect ophthalmoscopy, and slit-lamp biomicroscopy using a 90 D Volk lens. Optical coherence tomography (OCT) and fundus fluorescein angiography (FFA) was done whenever it was necessary. All cases were then examined for the presence or absence of diabetic retinopathy.

Those cases with fundus showing features of diabetic retinopathy were graded into five classes (mild NPDR, moderate NPDR, severe NPDR, very severe NPDR, PDR) based on early treatment diabetic retinopathy study (ETDRS) classification. Fasting blood glucose levels and postprandial blood glucose levels were assessed in all the cases from the blood sample collected from the antecubital vein. This was done to diagnose new cases and also to get a crude estimate of the diabetic control. Criteria for the diagnosis of diabetes was according to the American Diabetic Association [9], which is fasting blood glucose >126 mg/dL (7.0 mmol/L) or two hours postprandial blood glucose of >200 mg/dL (11.1 mmol/L). Random mid-stream urine samples were collected in autoclaved, dry corked glass bottles from patients after one hour of rest before collection in the morning. Quantitative assessment of urine albumin levels was done by turbidometric immunoassay using a fully automated analyser. Depending on the urinary albumin excretion (ug/mg creatinine), three categories were made - normoalbuminuria < 30, microalbuminuria 30-299 and macroalbuminuria > 300.

Statistical analysis

Data were coded and recorded in the MS Excel (Microsoft, Redmond, WA, USA) spreadsheet program. SPSS version 23 (IBM Corp., Armonk, NY, USA) was used for data analysis. Descriptive statistics were elaborated in the form of means/standard deviations and medians/interquartile ranges (IQRs) for continuous variables and frequencies and percentages for categorical variables. Data were presented graphically wherever appropriate for data visualization using histograms/column charts for continuous data and bar charts/pie charts for categorical data. For comparing continuous data, we used paired t-test to check for the significance between the two methods used. P-value < 0.05 was considered to be statistically significant.

## Results

All patients were between 40 and 85 years of age. The mean age of the cases was 60.03 ± 0.8 years.

Out of the 250 patients, 140 (56.0%) were males and 110 (44.0%) females.

In our study, the duration since diagnosis of diabetes mellitus ranged from 1-25 years. Among the 250 patients, 151 patients (87.2%) had no retinopathy in less than five years since diagnosis, while 22 patients (12.71%) had retinopathy. In patients with a duration of 6-10 years, 14 patients (27.45%) had no retinopathy, while 37 patients (72.54%) had retinopathy. In patients with a duration of 11-15 years, 20 patients had retinopathy. Similarly, in a group of more than 15 years, all six patients had retinopathy. Thus, retinopathy was seen in all patients who had diabetes for more than 10 years. The association between the severity of diabetic retinopathy with the duration of diabetes was significant (p =< 0.005) (Table 1).

**Table 1:** Association between grades of Diabetic Retinopathy and duration since diagnosis (in years)

Grades of Diabetic Retinopathy		Duration since diagnosis (in years)				P value
		Less than or equal to 5	6-10	11-15	More than 15	
No retinopathy	No.	151	14	0	0	<0.005
	%	87.28%	27.45%	0%	0%	
Mild NPDR	No.	12	16	1	0	
	%	6.93%	31.37%	5%	0%	
Moderate NPDR	No.	10	14	3	0	
	%	5.78%	27.45%	15%	0%	
Severe NPDR	No.	0	3	4	0	
	%	0%	5.8%	20%	0%	
Very severe NPDR	No.	0	1	4	2	
	%	0%	1.96%	20%	33.3%	
PDR	No.	0	3	8	4	
	%	0%	5.8%	40%	66.6%	
Total	No.	173	51	20	6	

One hundred seventy-two (68.8%) patients were hypertensives, and 78 (31.2%) patients were non-hypertensives. Among the hypertensive patients, 66.7% had diabetic retinopathy, and among the non-hypertensive patients, 19.2% had diabetic retinopathy (Table 2).

**Table 2:** Systemic hypertension and grades of retinopathy

	Hypertensives	Non-Hypertensives	Total
No retinopathy	26	139	165
Mild NPDR	11	18	29
Moderate NPDR	18	9	27
Severe NPDR	3	4	7
Very severe NPDR	5	2	7
PDR	15	0	15
Total	78 (31.2%)	172 (68.8%)	250 (100%)

Among the 250 patients, 165 (66.0%) patients had no retinopathy, 29 (11.6%) patients had mild NPDR, 27 (10.8%) patients had moderate NPDR, seven (2.8%) patients had severe NPDR, seven (2.8%) patients had very severe NPDR and 15 (6.0%) patients had PDR. One hundred and eighty-four (73.6%) patients were on oral hypoglycemic, 50 (20.0%) patients were on insulin, and 16 (6.4%) patients were not on any form of treatment. One hundred and fifty-one (60.4%) had normoalbuminuria, 78 (31.2%) had microalbuminuria, and 21 (8.4%) patients had macroalbuminuria. In this study, diabetic retinopathy was present in 9.27% of the cases in the normoalbuminuria group. The proportion of cases with retinopathy in the microalbuminuria group was 64.1%. It was also found that all of the cases within the macroalbuminuria group had retinopathy.

In the no retinopathy group, 83.03% had normal albumin levels in urine, whereas 16.96% had microalbuminuria. In the mild NPDR group, 37.94% of the cases had normoalbuminuria, and 62.06% of the cases had microalbuminuria. In the moderate NPDR group, 11.1% of the cases had normoalbuminuria, and 88.8% of the cases had microalbuminuria. None of the patients in the above-mentioned three groups had macroalbuminuria. In the severe NPDR group, 57.14% of the cases had microalbuminuria, while 42.86% had macroalbuminuria. In the very severe NPDR group, 42.86% of the cases had microalbuminuria, and 57.14% of the cases had macroalbuminuria. In the PDR group, only 6.6% of the cases had microalbuminuria, and the rest, 93.3% of the cases had macroalbuminuria (Table 3).

**Table 3:** Association between Grades of Diabetic Retinopathy and Urine albumin Excretion

Grades of Diabetic Retinopathy		Urine albumin Excretion			Total	P value
		Normal	Micro	Macro		
No retinopathy	No.	137	28	0	165	<0.001
	%	83.03%	16.96%	0%	100%	
Mild NPDR	No.	11	18	0	29	
	%	37.93%	62.06%	0.0%	100%	
Moderate NPDR	No.	3	24	0	27	
	%	11.1%	88.8%	0%	100.0%	
Severe NPDR	No.	0	4	3	7	
	%	0%	57.14%	42.86%	100.0%	
Very severe NPDR	No.	0	3	4	7	
	%	0%	42.86%	57.14%	100.0%	
PDR	No.	0	1	14	15	
	%	0%	6.6%	93.3%	100.0%	
Total	No.	151	78	21	250	

## Discussion

In the present study, 250 patients having type-II diabetes mellitus between 40 and 85 years were studied. Our study is in concordance with the other studies; the distribution of cases with respect to gender was even with a male to female ratio [M: F] of 56:44, and two of the previous studies, however, had a male predominance [10-13]. The difference concerning the sex distribution was not statistically significant. The patients were categorized concerning the presence or absence of diabetic retinopathy. In the group having retinopathy, patients were subcategorised depending on the severity/grade of retinopathy.

Patients with more than 10 years of diabetes accounted for 10.4% in the present study. The association between the grade of diabetic retinopathy and duration since diagnosis was significant (p = <0.005). A similar significant difference was found in studies [11,14]. Retinopathy was seen in all patients who had diabetes for more than 10 years. The association between the severity of diabetic retinopathy with the duration of diabetes was significant (p =< 0.005). The present study, akin to other studies, supports the observation that diabetic retinopathy severity increases with the duration of diabetes [15-17]. Hypertension commonly co-exists in diabetics and is considered an important confounding factor in the vascular complications of diabetes. In the present study, 31.2% of the cases were hypertensives. The proportion of hypertensives who had co-existing diabetic retinopathy was 68.0%. 

Limitations in the present study

The referral of uncontrolled diabetics to the tertiary centre for further management allows the possibility of selection bias to creep into the study. This gives future scope for a population-based study rather than a hospital-based study. The exact control of diabetes was not known since HbA1c was not tested. A study with a larger sample size can be conducted for a more conclusive result.

## Conclusions

In this study, microalbuminuria was associated with all grades of retinopathy with skewing towards the lower grades of diabetic retinopathy; a proportion of diabetics without retinopathy also had microalbuminuria, while macroalbuminuria was associated only with those patients who had either severe NPDR, very severe NPDR, or PDR. However, it was found that the occurrence of macroalbuminuria is significantly higher in severe NPDR, very severe NPDR, and PDR. Thus, this study reinforces the observation that there is a strong association between albuminuria and diabetic retinopathy in type-II diabetes.
